# Studying a disease with no home - lessons in trial recruitment from the PATCH II study

**DOI:** 10.1186/1745-6215-11-22

**Published:** 2010-03-02

**Authors:** Kim S Thomas, the UK Dermatology Clinical Trials Network's PATCH study group

**Affiliations:** 1 Centre of Evidence Based Dermatology, University of Nottingham, King's Meadow Campus, Nottingham NG7 2NR, UK

## Abstract

**Background:**

Cellulitis is a very common condition that often recurs. The PATCH II study was designed to explore the possibility of preventing future episodes of cellulitis, with resultant cost savings for the NHS. This was the first trial to be undertaken by the UK Dermatology Clinical Trials Network. As such, it was the first to test a recruitment model that involved many busy clinicians each contributing just a few patients.

**Methods:**

A double-blind randomised controlled trial comparing prophylactic antibiotics (penicillin V) with placebo tablets, for the prevention of repeat episodes of cellulitis of the leg. Primary outcome was time to subsequent recurrence of cellulitis.

**Results:**

The PATCH II study was closed to recruitment having enrolled 123 participants from a target of 400. Whilst the recruitment period was extended by 12 months, it was not possible to continue beyond this point without additional funds. Many factors contributed to poor recruitment: (i) changes in hospital policy and the introduction of community-based intravenous teams resulted in fewer cellulitis patients being admitted to hospital; ii) those who were admitted were seen by many different specialties, making it difficult for a network of dermatology clinicians to identify suitable participants; and iii) funding for research staff was limited to a trial manager and a trial administrator at the co-ordinating centre. With no dedicated research nurses at the recruiting centres, it was extremely difficult to maintain momentum and interest in the study. Attempts to boost recruitment by providing some financial support for principal investigators to employ local research staff was of limited success.

**Discussion:**

The model of a network of busy NHS clinicians all recruiting a few patients into large clinical studies requires further testing. It did not work very well for PATCH II, but this was probably because patients were not routinely seen by dermatologists, and recruitment took place prior to research support being available through the Comprehensive Clinical Research Network (CCRN). There is a balance to be struck between asking a lot of centres to recruit just a few patients, and asking a few centres to recruit a lot of patients. Giving modest funds to principal investigators to buy local research nurse time did not work well, probably because too little research time was bought, and it was difficult to separate research tasks from the nurses existing clinical duties. National research infrastructure networks such as the Comprehensive Clinical Research Network will overcome many of the problems encountered in the PATCH II trial.

**Trial Registration:**

The trial registration number is ISRCTN03813200.

## Background

Cellulitis of the leg is a painful and potentially serious infection of the skin and subcutaneous tissue. It is a very common condition, which was highlighted by Patricia Hewitt MP in her report to the House of Commons on ways of reducing emergency care admissions [1]. This report found that cellulitis was amongst the top ten reasons for admission to hospital, accounting for over 45,000 emergency admissions per year (at a total cost of £87 million). Cellulitis is an acute condition that requires rapid assessment and treatment. Patients are treated by many specialties including emergency care, general practitioners, general medicine, surgery, tissue viability and dermatology [2]. With so many specialties involved, the identification of potential participants for a clinical trial involving cellulitis patients can be difficult.

The PATCH study was one of the first to be developed and run through the UK Dermatology Clinical Trials Network (UK DCTN). The UK DCTN has charity status, and was established about seven years ago in order to conduct high-quality randomised controlled trials (RCTs) that answer questions of importance to clinicians and patients. It is now a collaborative network of just over five-hundred dermatologists, dermatology nurses, health services researchers and patients throughout the UK and Southern Ireland. The Network seeks to answer important clinical questions that have a direct impact on day-to-day clinical practice, and which are unlikely to be answered by commercially-driven pharmaceutical research.

The structure and remit of the UK DCTN was initially modelled on the work of the UK Childhood Cancer Study Group. However, as a result of the recommendations published by the Department of Health in 'Best Research for Best Health' [3] there has recently been a substantial investment in clinical trial infrastructure in the UK. Five new topic specific research networks, a primary care research network and a comprehensive clinical research network have been established [4]. The work of the UK DCTN is supported through the Comprehensive Clinical Research Network, which is responsible for all disease areas not covered by one of the topic specific research networks. Through this infrastructure, research nurses are now in place to assist with recruitment into both commercial and non-commercial trials in the UK. However, these resources were not available at the time that we were recruiting into the PATCH study.

## Methods

### Recruitment Model

The PATCH II trial was designed to answer the question; "Does low dose penicillin taken after an episode of cellulitis of the lower leg reduce the risk of further episodes?" This was a double-blind, placebo controlled randomised controlled trial comparing low-dose penicillin V with placebo. The primary outcome for the study was time to repeat episode of cellulitis of the leg. Ethical approval for the study was granted by Nottingham NHS Research Ethics Committee 2 (Ref: 06/Q2404/22) and the study was registered on the Controlled Clinical Trials website (ISRCTN34716921).

## Results

Initial sample size calculations suggested that 400 participants were required for the study, and recruitment was intended to take place over a 12-month period. However, after 24 months of recruitment, the study was closed to new recruits with just 123 (31%) participants enrolled. The funders were unable to support our request for a funded extension period to reach our planned recruitment target.

Despite being a relatively common condition, recruitment for the study took place in a large number of UK centres (25+), each of which was to recruit 1-2 participants per month. This was deemed to be the best approach for two reasons: i) it provided the opportunity for ALL members of the newly formed network to contribute to the study, and ii) the study was funded through a medical charity (the BUPA Foundation), which meant that insufficient funds were available to employ dedicated research nurses to work on the study. The lack of funds to employ dedicated research nurses necessitated the involvement of many investigators. To minimise the burden on recruiting investigators, participants were followed up through telephone interviews conducted by staff at the co-ordinating centre, and were not required to return to see the recruiting investigator unless they had a repeat attack of cellulitis.

One of the reasons for choosing this study as the first to be conducted through the UK Dermatology Clinical Trials Network was that at the time (2006/07), patients were generally admitted to hospital for a period of up to ten days, giving ample time for the identification of suitable participants, and for obtaining informed consent. The policy of routinely admitting cellulitis patients was then changed with the introduction of community-based IV teams (that offer intravenous antibiotics at home or in a primary care setting), and the increased use of day-treatment units to treat cellulitis patients [5,6]. This coincided with the start of the trial and contributed to some of the recruitment difficulties that the team faced. Similar issues have been reported by other investigators where changes in NHS practice and restructuring have impacted on their ability to recruit [7].

## Discussion

### Was the study design at fault?

During the development of this trial, all best practices were followed and every effort was made to ensure that the study was well-designed and appropriate (Table 1). The research question continues to be an important clinical question that has been identified as a research gap in recent cellulitis guidelines [8], and continues to be well supported by the clinical community.

**Table 1:** Factors that may predict recruitment success[10].

Relevant factors	PATCH II
Quality of Study team/multi-disciplinary team	Experienced multi-disciplinary team including dermatologists, trialists, statistician and health economist. Independent Chair of Steering Committee and independent Data Monitoring Committee.
Involvement of a Clinical Trials Unit	Yes - Nottingham Clinical Trials Unit (CTU)
Trial Manager	Yes - based at co-ordinating centre
Local recruitment co-ordinators	No - this was a major weakness for PATCH II. Local research nurses, with dedicated time to work on the study could have made a real difference to recruitment.
Feasibility work	Twelve-month feasibility study conducted prior to starting trial.
Peer reviewed study protocol	Protocol peer reviewed by UK DCTN and Nottingham CTU
Simple study design	Simple parallel group study (but complicated by recruitment into a similar study that was being run in parallel).
Service user input	Focus group discussions as part of feasibility study & through discussion with the Lymphoedema Support Network
Important research question with support of the clinical community	Identified as a priority trial by the UK Dermatology Clinical Trials Network
Drug trial/intervention only available in study	Yes. Some may have felt that prophylactic antibiotics were only available to them within the trial, although this depended very much on local practice of the treating physician.
Appropriately funded	Limited funding available due to charity as the source of funding.

### Was our model of recruitment wrong?

The model of many clinicians all contributing just a few patients into very simple and low impact trials alongside normal clinical duties is a model that has shaped the development of the UK DCTN, and is critical for determining the success of such a network. Despite the difficulties recruiting into the PATCH study, it is important that work continues to establish if this model would be effective for less common conditions that are routinely seen by dermatologists only, or whether the model is fundamentally flawed.

Tasks allocated to the recruiting physicians were kept to an absolute minimum and consisted of: i) identifying the patients; ii) confirming the diagnosis/suitability of the participant to be randomised into the study, iii) taking informed consent and iv) prescribing the trial drugs. All other tasks were delegated to staff at the co-ordinating centre, including randomisation of the participants, telephone follow-up, reporting of adverse events and data checking. Such an approach was deliberately chosen in order to make best use of clinicians' time, and others have reported that centralising processes such as replacing telephone randomisation with postal randomisation does not compromise recruitment success [9].

Crucially for the PATCH II study, it was the initial identification of potential participants that was difficult and time consuming for the clinicians. In order to identify patients for the study, investigators had to search discharge coding, accident & emergency coding and to visit wards and departments where cellulitis patients were regularly treated. This process was time-consuming, and was clearly a large commitment for clinicians to fit into their already busy workload. The Strategies for Trials Enrolment and Participation Study (STEPS) highlighted some of the key factors that might predict successful recruitment into a trial. Not having paid trial co-ordinators on-site was highlighted as being one of the most important factors [10], which proved to be a crucial stumbling block for the PATCH II study.

### Providing help for centres to identify patients

In an attempt to improve recruitment, additional UK DCTN funds were used to buy research support time at 12 of the recruiting centres. Money was provided for between 3 and 18 months (median 6 months), and was used to pay overtime to existing clinic or administrative staff. The new staff were asked to help the principal investigators to identify suitable participants for the trial, thus reducing the administrative burden for the clinical team. Overall, nine research nurses, and three administrative staff were employed for between two to four hours per week. Whilst these additional funds provided a small increase in recruitment, this was a lot less successful than we had hoped. Feedback from centres suggests that this was because they were unable to ring-fence sufficient time to work on the study, and with just a few extra hours each week, they failed to gain a sense of ownership of the trial. These factors were felt to be more important than whether the additional support was provided by a nurse or by administrative staff.

Throughout the study, a variety of techniques were used in order to boost recruitment, with varying degrees of success. These are summarised in Table 2. Similar strategies have been reported by other trialists [10], although the impact of these on trial accrual is often difficult to establish.

**Table 2:** Strategies employed to boost recruitment

Strategies with good impact	
Pay sites (for nurse time or administrative support)	Some success, but difficult to ring-fence time
Reduce paperwork	Good feedback from investigators
Training workshops	Good for boosting morale, temporary increase in recruitment
Increase number of sites	Limited success, lots of additional work
Advertise in local papers/radio	Good response, but limited by lack of funds
Amend protocol/procedures	Some success
Regular contact between co-ordinating centre and site	Some success, but difficult to contact doctors

**Strategies with little impact/unsure of impact**	

Regular newsletter (to recruiting sites and participants)	Unsure
Website	Unsure
Small gifts to top recruiters	Unsure
Articles in journals/support group newsletters	Unsure
Letters of encouragement from CI and PI	Limited impact
Expand recruitment into primary care	Delays in approvals - not implemented in time

## Conclusions

### Lessons learned

We are currently recruiting to a second cellulitis study (PATCH I) and are concentrating our efforts on working with the Comprehensive Local Research Networks (CLRNs) to support centres with dedicated research nurses. These research nurses will not be distracted by other clinical duties and so should be more able to identify patients for the study. Recruitment into PATCH I is proceeding well with 186 (72%) out of a target of 260 participants having been successfully recruited at the time of writing this paper. Similar success in boosting recruitment through the Comprehensive Local Research Network has been reported elsewhere [11]. We are also building a sense of ownership and shared responsibility amongst the trial staff by holding regular training sessions and telephone-conferences (especially between research nurses). These provide a forum for sharing best practices and for learning about successful recruitment strategies in the different centres.

With the advent of the EU Clinical Trials Directive and improved standards for the conduct of clinical trials in the UK, it is increasingly difficult to deliver high quality non-commercial clinical trials without substantial funding. The majority of NIHR Health Technology Assessment funded trials now cost between £600,000 - £1,200,000 http://www.ncchta.org/project, and such levels of funding are simply not available from the majority of charitable bodies that fund dermatology research. The PATCH II trial award was for £190,000 and the charity supporting our study was not able to support a grant extension. A review of trials funded by the Medical Research Council and the Health Technology Assessment programme showed that less than a third of a cohort of 114 trials recruited to target, and more than a third of them were granted funded trial extensions [10]. The role of charities in being sole funders of clinical trials has to be questioned.

The Network now has two further studies underway in bullous pemphigoid and pyoderma gangrenosum [12], both of which are well funded through NIHR funding bodies [13]. These studies are multi-centre (40-50 recruiting sites) and will use the same recruitment model as PATCH II (i.e. many recruiting centres all contributing a small number of patients). However, there are three significant differences between these studies and the PATCH II study; i) both studies are recruiting patients with conditions that are routinely treated by dermatologists; ii) the level of funding for these studies is over four times that which is available through charitable sources; and iii) CLRN research nurses will be available to support the investigators in many of the centres. We will also follow the example of other successful investigators with experience of running large multi-centre trials, such as the UK Collaborative Trial of Ovarian Cancer Screening study [14]. This study successfully recruited more than 200,000 women into a randomised controlled trial and recommended a highly proactive management style; a willingness to tailor solutions to individual sites; automation of processes and web-based trial management systems; the use of training videos; and organisation of regular training meetings in order to facilitate group discussion amongst co-investigators and other members of the study team.

For the future the UK DCTN will need a balanced and varied portfolio of studies. A strategic decision has been made to develop trials of three keys types: i) trials that rely on many investigators each providing small numbers of participants with an uncommon condition (e.g. bullous pemphigoid; ii) trials of more common conditions that can be done in a small number of centres with dedicated research support (e.g. acne and eczema); iii) trials involving specialty groups that have a particular interest in a disease or process (e.g. trials involving dermatology surgeons).

## Competing interests

The authors declare that they have no competing interests.

## Authors' contributions

The writing team comprised: Kim Thomas (lead applicant), Joanne Chalmers (senior trials manger), Katharine Foster (trial manager), Hywel Williams (chief investigator).
